# Dr. T. B. Hamlin

**Published:** 1874-07

**Authors:** 


### OBITUARY.

---

*The Late Dr. Hamlin.*—A meeting of the dentists of Nashville was called at the office of Dr. Ross, for the purpose of paying a last tribute of respect to the memory of Dr. T. B. Hamlin, who died at his home near Edgefield Junction.

Dr. Ross was appointed Chairman of the meeting, and Dr. Noel Secretary.

A committee of three, composed of Drs. Morgan, King and Cobb, were appointed to draft suitable resolutions.

After a short consultation the committee presented the following, which were adopted.

Whereas, God in His Providence has taken to Himself our highly esteemed friend and former colaborer, Theodore Burnam Hamlin, D. D. S., and believing the Almighty Father doeth all things well, and that his providence are always wise and good, we bow with humble submission to his Divine will.

Resolved, that we here record our high appreciation of his character, both as a Christian gentleman, and a professional man. And we would call to mind his marked energy of character, his concentration of purpose, and his lofty aspirations for perfection as worthy of imitation.

Resolved, that we cherish the memory of his life, and especially of his professional life, and hold it up as worthy of imitation by our professional brethren.

Resolved, that in his death, his family lose a kind and affectionate husband and father, his Church an upright, zealous member, and the Masonic Fraternity one of its brightest jewels. But our brother so lived that

> "When the summons came to join
> The innumerable caravan that moves
> To quarry the mysterious realms, where each shall take.
> His chamber in the silent halls of death,
> He went, not like the slave at night,
> Scourged to his dungeon, but, sustained and soothed
> By an unfaltering trust in God, he approached his grave
> Like one that draws the drapery of his couch
> About him, and lies down to pleasant dreams."

Resolved, that we will attend his funeral services in a body.

Resolved, that a copy of these proceedings be sent our daily papers for publication, and that a copy be furnished the family of the deceased.

Resolved, that a copy of these resolutions be furnished all the Dental Journals for publication.

Dr. Hamlin was born on June 24th, 1810, in Windom, New York. He was left an orphan at an early age without means, and almost without friends or kindred. At eighteen years of age he was foreman in the largest watchmaker's establishment in Albany, and perhaps in the United States, where his attention was first directed to dentistry. He removed to Wytheville Va., about 1834 or 1835. At this time we find him taking an active part in the organization of Dental societies. He assisted in the organization of the Virginia Dental Society, the first of its kind in America or in the world, so far as is known to the profession. He removed to Tuscumbia, Ala., about the year 1845, and thence to Nashville in 1847, where he did a large business in connection with Dr. Morgan, until the close of 1858, at which time his health failed, and he went into the nursery business in connection with bee culture. In the latter business he was authority, having published a practical and highly-prized work on bee culture a few years since. He was also Vice-President of the National Association of Apiarians at the time of his death.

Dr. Hamlin was a member of the Presbyterian Church and an ornament to the Masonic fraternity. He was a member of indomitable energy, and for him to conceive, was to execute.

---

